# Reevaluating the role of fungal blood cultures in clinical practice

**DOI:** 10.1017/ash.2026.10818

**Published:** 2026-09-01

**Authors:** Logan Brock, Anastasia Engeleit, Kathryn E. DeSear, Pooja Gurram, Tristan R. Grams, Lisa Vuong

**Affiliations:** 1 Department of Pharmacy, UF Health Shands Hospital, USA; 2 Department of Pharmacotherapy and Translational Research, College of Pharmacy, University of Florida, USA; 3 Division of Infectious Diseases and Global Medicine, College of Medicine, University of Florida, USA; 4 Department of Pathology, Immunology, and Laboratory Medicine, College of Medicine, University of Florida, USA

## Abstract

In this retrospective study, fungal blood cultures rarely detected fungal pathogens not found in paired standard blood cultures, with added findings in only 0.4% of cases. Given the low diagnostic yield and high cost, results support reserving fungal blood cultures for select high-risk patients to improve diagnostic stewardship.

## Introduction

Sepsis is a major healthcare burden and timely management is critical to improving outcomes.^1^ Quality measures such as the Centers for Medicare and Medicaid Services sepsis bundle emphasize early broad-spectrum antibiotics and blood culture collection.^2^ While the efficacy of this bundle has come under scrutiny due to limited randomized trials and concerns regarding overtreatment, clinical practice still emphasizes initiating broad-spectrum antibiotics together with an infectious workup.^3^ At our institution, fungal blood cultures are sometimes ordered alongside standard blood cultures, particularly in patients with risk factors for invasive fungal infections.^4^ However, the diagnostic value of routinely including fungal blood cultures in this setting remains uncertain.

Contemporary standard blood cultures, particularly those processed using automated continuous monitoring systems (eg, BD BACTEC™), can detect *Candida* species with reported sensitivities ranging from 63% to 83% and a median time-to-positivity of 2–3 days.^5^ Since multiple sets of standard blood cultures are often obtained, the added diagnostic value of fungal blood cultures may be limited. Fungal blood cultures are typically collected using fungal-specific media (eg, BD Myco/F Lytic medium) or, historically, via isolator-based systems, which have recently been discontinued in the United States. Use of Myco/F Lytic blood culture bottles provide enhanced recovery of fungi by incorporating saponin-based lytic agents that liberate intracellular organisms and by using media optimized for fungal growth with extended incubation. These features improve detection of slow-growing and fastidious fungi, including dimorphic fungi and select yeasts, that may not be recovered in routine aerobic or anaerobic blood culture bottles.^6^ Accordingly, Myco/F Lytic bottles occupy a narrowly defined diagnostic niche yet are frequently overutilized in routine practice, where indiscriminate use is inappropriate given the availability of alternative fungal diagnostics that are often more timely and sensitive.^7^

Fungal blood cultures can also financially impact patients and the healthcare system. While the material cost is low at approximately $13, the specialized labor required for processing leads many commercial laboratories to charge between $99 and $119 per culture. Stanley et al estimated the patient cost at $110.^8^ Given the cost and uncertain added value; this study was conducted to evaluate the clinical utility of collecting fungal blood cultures alongside standard blood cultures.

## Methods

A retrospective, cross-sectional study was performed at UF Health Shands Hospital, a 1,149-bed academic teaching hospital. Adult patients were included if they had initial fungal and standard blood cultures collected within 24 hours of each other between 1/1/2023 and 12/31/2024. The primary outcome was the detection of fungal growth in a fungal blood culture without growth in the paired standard blood cultures. Secondary outcomes evaluated time-to-positivity, all-cause in-hospital mortality, 30-day mortality, and the cost of detecting an additional fungus through usage of fungal blood cultures. Fungal blood cultures were collected on BD Myco/F Lytic medium and monitored for growth for 28 days via the BD BACTEC™ blood culture system. Standard blood cultures were collected on BD standard aerobic and anaerobic medium and incubated for 5 days. Analyses were performed using JMP®, version 18.0.2 (SAS Institute, Cary, NC). Descriptive statistics were used to summarize categorical data. χ^2^ and Mann-Whitney *U* tests were used as appropriate. All *P*-values were 2-tailed, and *P* < .05 was considered statistically significant. This study was approved by the Quality Improvement Project Registry at the University of Florida.

## Results

**Figure 1.**
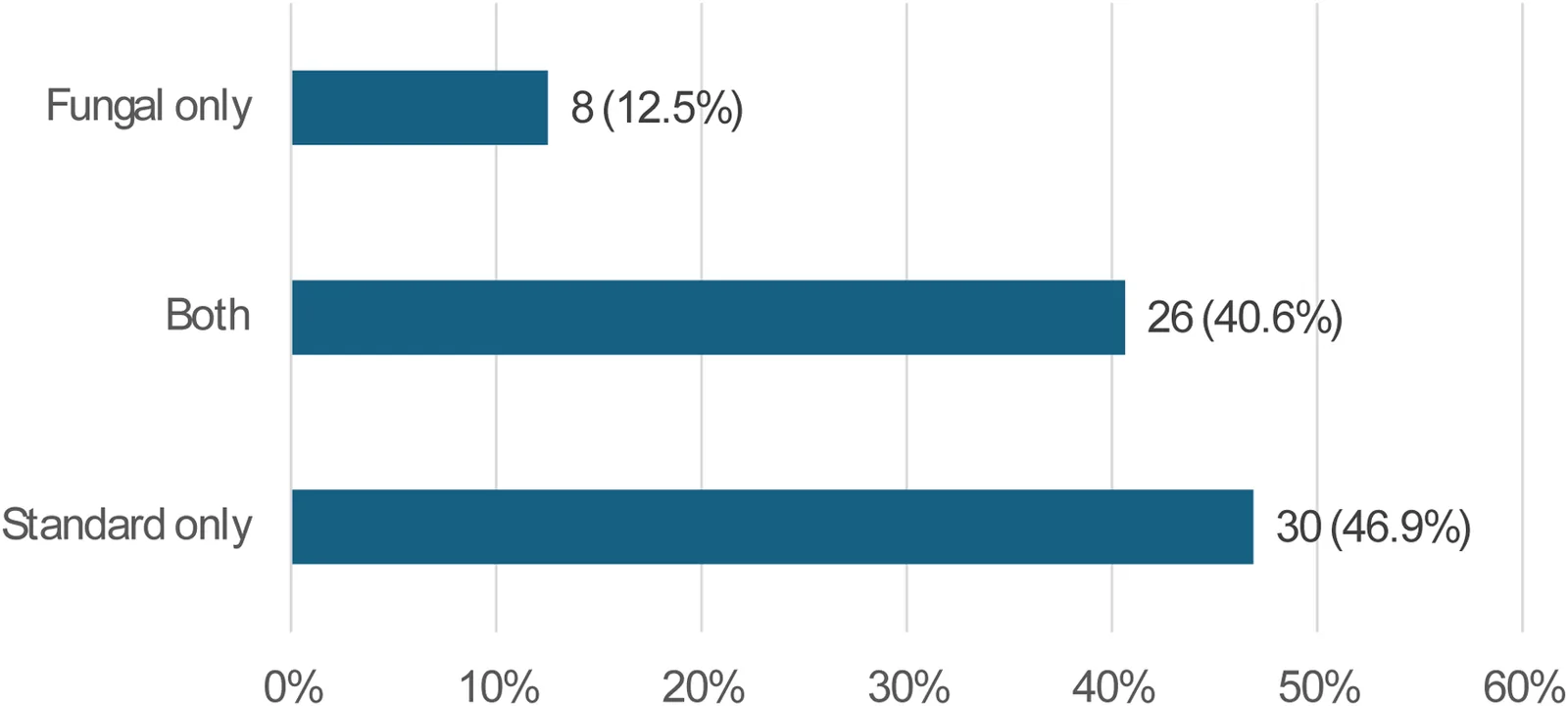
Distribution of fungal growth by blood culture type (n = 64).

Of 2,449 collections screened, 2,073 paired initial fungal and standard blood cultures met inclusion criteria. Baseline characteristics are displayed in Table 1. Of the 2,073 paired blood cultures, 64 (3.1%) yielded fungal growth in at least one culture type, while the remaining 2,009 (96.9%) revealed no fungal growth in either culture type. Growth in the fungal culture but not standard cultures occurred in 8/2,073 (0.4%) pairs (Figure 1).

**Table 1.** Baseline characteristics Table 1 long description.

Characteristic	Total, N = 2,073
Age (years), median (IQR)	60 (46–69)
Female, n (%)	938 (45)
Race, n (%)	
White	1,428 (69)
Black	410 (20)
Multiracial	24 (1)
Asian	23 (1)
Other	188 (9)
Weight (kg), median (IQR)	76 (63–92)
BMI (kg/m^2^), median (IQR)	26 (22–31)
Blood cultures drawn in ICU, n (%)	1,234 (60)
Days from admission to blood culture collection, median (IQR)	2 (0–10)

IQR, interquartile range; BMI, body mass index; ICU, intensive care unit.

In a subgroup analysis of the 64 paired cultures that detected fungal growth in any culture type, 26 (40.6%) were from both culture types and 30 (46.9%) were only from standard blood cultures. *Candida* species accounted for 60 pairs (93.8%). The other four were *Cryptococcus*, *Histoplasma*, *Lodderomyces*, and *Malassezia* species. *Cryptococcus* and *Lodderomyces* species were recovered from both media types, *Histoplasma* species grew exclusively in fungal blood culture media, and *Malassezia* species grew only from standard blood culture media.

Using commercially available estimates, a cost of $99 was used per fungal blood culture bottle. The two-year period that resulted in 8/2,073 (0.4%) positive pairs detected in only fungal blood culture media was used for this calculation. This corresponds to an annual institutional cost of approximately $\frac{2,073\;paired\;blood\;culture\;bottles\;\times \;\$99}{2\;years}=\$102,613.50$ to detect the additional 4 cases per year.

Among fungi recovered in both media types, fungal blood cultures had a significantly longer median time to positivity than standard cultures (48 h [interquartile range, IQR, 41–93] vs 33 h [IQR, 28–42]; *P* < .001). All-cause in-hospital mortality occurred in 361 (17.4%) encounters, and 30-day mortality occurred in 339 (16.4%) encounters.

## Discussion

Empirically paired fungal blood cultures rarely identify fungal pathogens not detected by standard blood cultures. Fungemia was uncommon and occurred at 3% of paired cultures, the majority (94%) of which were due to *Candida* species.

Another major finding was that this study largely recovered fungal growth only in standard blood cultures. One explanation for this is that modern standard aerobic blood culture media are highly optimized and perform exceptionally well for the recovery of *Candida* species, which represents the vast majority of fungemia cases. Specialized fungal blood culture media are typically only advantageous for the recovery of dimorphic fungi, molds, or fastidious yeasts, which are comparatively rare. Our findings align with Babb et al, who assessed fungal detection in critically ill patients. In their analysis, fungal growth was recovered in 42/1094 (3.8%) of standard blood cultures and 4/523 (0.76%) of fungal blood cultures. Notably, all fungal species recovered from standard cultures in their study were *Candida*.^9^

Only one blood culture pair in our study yielded *Histoplasma* species, with growth observed only in the fungal blood culture. This highlights one possible niche of fungal blood cultures, as described by Herrera et al.^7^ At our institution, *Histoplasma* infections are rare. Fungal blood cultures may still have added utility in more endemic areas or in immunocompromised patients with specific risk factors. Alternatively, other diagnostic methods, such as urine antigen testing, may offer both faster turnaround and higher sensitivity.^10^

Limitations to this study included its retrospective nature and single center setting, which may limit generalizability and not capture institutional or regional differences in fungal epidemiology. This study was not designed to evaluate the impact of fungal blood cultures on patient outcomes. Risk factors for invasive fungal infections such as intensive care unit stay, central line use, and immunosuppression were not assessed. However, given the low diagnostic yield and significant cost burden shown in this study, it may be reasonable to restrict fungal blood culture use to those with known risk factors and high risk of non-*Candida* infections. Future areas of research could include fungal blood culture diagnostic stewardship, and prospective, pragmatic approaches with cost-effectiveness analyses.

## Research transparency and reproducibility

None to declare.

## Table 1. Long description

A table displaying baseline characteristics of a study population. The table has 13 rows and 2 columns. The first column lists characteristics such as age, gender, race, weight, BMI, blood cultures drawn in ICU, and days from admission to blood culture collection. The second column provides the corresponding total values or percentages. Row 1: Age (years), median (IQR), Total: 60 (46-69). Row 2: Female, n (%), Total: 938 (45). Row 3: Race, n (%), Total: White, 1,428 (69); Black, 410 (20); Multiracial, 24 (1); Asian, 23 (1); Other, 188 (9). Row 4: Weight (kg), median (IQR), Total: 76 (63-92). Row 5: BMI (kg/m^2), median (IQR), Total: 26 (22-31). Row 6: Blood cultures drawn in ICU, n (%), Total: 1,234 (60). Row 7: Days from admission to blood culture collection, median (IQR), Total: 2 (0-10).

Navigate back to Table 1..

## References

[1] La Via L , Sangiorgio G , Stefani S , et al. The global burden of sepsis and septic shock. Epidemiologia (Basel) 2024;5:456–478. 10.3390/epidemiologia5030032 PMC11348270 39189251

[2] Motzkus CA , Lilly CM. Accountability for sepsis treatment: the SEP-1 core measure. Chest 2017;151:955–957. 10.1016/j.chest.2017.01.011 28483129

[3] Freund Y , Cancella de Abreu M , Lebal S , et al. Effect of the 1-h bundle on mortality in patients with suspected sepsis in the emergency department: a stepped wedge cluster randomized clinical trial. Intensive Care Med 2024;50:1086–1095. 10.1007/s00134-024-07509-1 38913098

[4] Thomas-Rüddel DO, Schlattmann P , Pletz M , Kurzai O , Bloos F. Risk factors for invasive candida infection in critically ill patients: a systematic review and meta-analysis. Chest 2022;161:345–355. 10.1016/j.chest.2021.08.081 PMC8941622 34673022

[5] Clancy CJ , Nguyen MH. Finding the “missing 50%” of invasive candidiasis: how nonculture diagnostics will improve understanding of disease spectrum and transform patient care. Clin Infect Dis 2013;56:1284–1292. 10.1093/cid/cit006 23315320

[6] Fuller DD , Davis TE Jr , Denys GA , York MK. Evaluation of BACTEC MYCO/F Lytic medium for recovery of mycobacteria, fungi, and bacteria from blood. J Clin Microbiol 2001;39:2933–2936. 10.1128/JCM.39.8.2933-2936.2001 PMC88263 11474016

[7] Herrera LN , Khodadadi R , Leal S , Kulkarni P , Pappas P , McCarty T. Clinical utility of routine use of fungal blood cultures. Am J Med 2023;136:514–517. 10.1016/j.amjmed.2023.01.037 36849070

[8] Stanley J , Guilfoose J , LaSala PR. A comparison of standard and fungal blood cultures in the detection of fungemia. Am J Infect Control 2011;39:E147–E148.

[9] Babb J , Clark A , Gaffney D , Abdelfattah K , Prokesch BC. Little utility of fungal blood cultures in surgical and burn intensive care units. Microbiol Spectr 2022;10:e0022822. 10.1128/spectrum.00228-22 PMC9431255 35762782

[10] Libert D , Procop GW , Ansari MQ. Histoplasma urinary antigen testing obviates the need for coincident serum antigen testing. Am J Clin Pathol 2018;149:362–368. 10.1093/ajcp/aqx169 29462251

